# Structure of Single-Chain Nanoparticles under Crowding Conditions: A Random Phase Approximation Approach

**DOI:** 10.1021/acs.macromol.3c01333

**Published:** 2023-11-03

**Authors:** Beatriz Robles-Hernández, Marina González-Burgos, Paula Malo de Molina, Isabel Asenjo-Sanz, Aurel Radulescu, José A. Pomposo, Arantxa Arbe, Juan Colmenero

**Affiliations:** † Donostia International Physics Center (DIPC), 20018 Donostia-San Sebastián, Spain; ‡ Centro de Física de Materiales/Materials Physics Center (CFM/MPC), 20018 Donostia-San Sebastián, Spain; § IKERBASQUE—Basque Foundation for Science, 48009 Bilbao, Spain; ∥ Jülich Centre for Neutron Science (JCNS) at Heinz Maier-Leibnitz Zentrum (MLZ), Forschungszentrum Jülich GmbH, 85748 Garching, Germany; ⊥ Department of Polymers and Advanced Materials: Physics, Chemistry and Technology, University of the Basque Country UPV/EHU, 20018 Donostia-San Sebastián, Spain

## Abstract

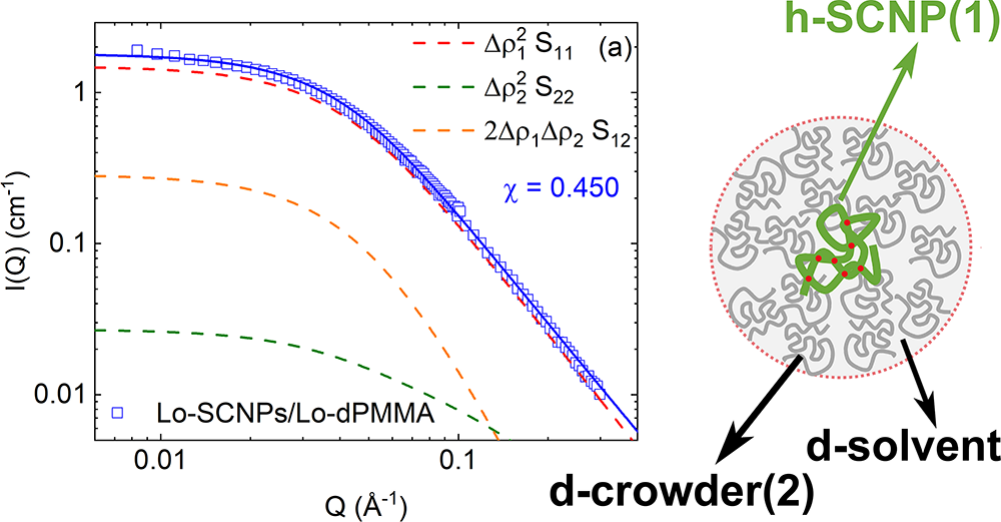

The conformation of poly(methyl methacrylate) (PMMA)-based single-chain nanoparticles (SCNPs) and their corresponding linear precursors in the presence of deuterated linear PMMA in deuterated dimethylformamide (DMF) solutions has been studied by small-angle neutron scattering (SANS). The SANS profiles were analyzed in terms of a three-component random phase approximation (RPA) model. The RPA approach described well the scattering profiles in dilute and crowded solutions. Considering all the contributions of the RPA leads to an accurate estimation of the single chain form factor parameters and the Flory–Huggins interaction parameter between PMMA and DMF. The value of the latter in the dilute regime indicates that the precursors and the SCNPs are in good solvent conditions, while in crowding conditions, the polymer becomes less soluble.

## Introduction

Macromolecules in biological systems *in vivo* are mostly in the cell’s crowded environment, where the high concentration of other molecules creates an effective nanoconfinement that can dramatically modify the biological function through changes in the structural conformation as well as in the dynamics with respect to diluted *in vitro* conditions.^1^ In particular, unstructured polymer chains—including intrinsically disordered proteins and unfolded coils—compress under crowding. In fact, the coil collapse is considered a necessary factor that drives protein folding.^2^ Aspects such as concentration, chain architecture, and intra- and intermolecular interactions will have an impact on the chain conformation. The understanding of the role of these factors requires structural characterization in crowded media, which is challenging given the high concentration and the intrinsic complexity in these samples.

Neutron scattering is the most suited technique to measure the form factor of small concentrations of labeled chains in the presence of large concentrations of other species. It measures both the structure and thermodynamic quantities, such as the Flory–Huggins interaction parameter. In dilute conditions, both small-angle X-ray and neutron scattering (SAXS/SANS) access the overall chain size as well as the fractal dimension inside the particle. However, in the semidilute regime, SANS and SAXS yield information about the chain correlations under full contrast conditions, *i.e.*, when all the macromolecules have a different scattering length density compared to the solvent. To probe the single-chain conformation in semidilute and concentrated solutions, appropriate contrast conditions are needed. This can be achieved for SANS by hydrogen/deuterium labeling. A strategy commonly used is masking out the contribution of one species in the mixture by contrast matching with the solvent. This is done by having a small amount of hydrogenated polymers in a mixture of deuterated polymers and deuterated solvent and assuming that the scattering signal arises exclusively from the hydrogenated polymer. However, to make sure that the scattering intensity yields the single chain form factor, the system must be in the so-called zero-average contrast conditions, which cancel out the intermolecular structure factor contribution to the total scattering.^3^

The chain size reduction of homopolymers in semidilute solutions is fairly well understood; theoretical approaches have led to the prediction that the radius of gyration scales with concentration as *R*_g_ ∼ *c*^–1/8^ in good solvent and this dependence was experimentally supported by SANS in homopolymer solutions.^3,4^ The same behavior was found in solutions in which the probe and the crowder are chemically different.^5^ In binary polymer mixtures where the effects of the polymer–polymer interactions are not negligible, these must be taken into account to quantify the chain compression.^6^ Intrinsically disordered proteins (IDPs), as they are unfolded chains, are also expected to contract in crowding media. However, SANS studies of IDPs in contrast-matched crowder solutions of diverse nature, from globular proteins to ramified polysaccharides, have yielded contradictory data. In some cases, a mild size reduction is reported^7^ and in others, even a size increase, attributed to a soft attraction with the crowders, which are of different chemical nature than the protein under study.^8^

Indeed, biological systems are heterogeneous and complex in nature, and the use of simplified models helps to bridge the gap between polymer solutions and biological mixtures. In particular, single-chain nanoparticles (SCNPs) can help address the essential question of the effect of crowding on the structure. SCNPs are single-stranded polymers partially collapsed *via* intramolecular bonding. To avoid unwanted intermolecular bonding, collapse is induced under high dilution. These nano-objects serve as simplified models for IDPs^9^ due to their internal structure and inherent polydispersity in size and morphology.^10^ General synthesis methods involve the functionalization of a linear precursor and subsequent intramolecular cross-linking.^11^ Each cross-link between two reactive functional sites of the chain generates an internal loop, and globally, the precursor chain reduces its size. The formation of long-range loops in good solvent conditions is less probable, and short-range loops are favored due to the self-avoiding conformation of the precursor, resulting in sparse, nonglobular SCNPs in solution.^12^ In addition, unlike globular proteins whose folding is driven by defined interactions, the collapse of synthetic SCNPs is the result of a stochastic process, leading not only to a less controlled compaction but also to a polydispersity of resulting topologies.^12,13^

The structural changes of SCNPs under crowding have been previously addressed using model SCNPs embedded in analogue linear crowder solutions.^14,15^ In one study, the effect of crowding of linear polystyrene (PS) of low- and high-molecular weights on the structure of PS-SCNPs showed that the crowders with different molecular weights have different effects: while long chains tend to impede their aggregation and lead to chain compression above their overlap concentration, short ones are found to mediate depletion interactions, leading to aggregation.^15^ In another study, the effect of poly(methyl methacrylate) (PMMA) on the structure of PMMA-based SCNPs was investigated by combining contrast variation SANS with molecular dynamics (MD) simulations. Results showed a crossover from unperturbed SCNP conformation in dilute conditions to a collapse starting when the total concentration reached the SCNP overlap concentration and continuing decreasing in size with increasing crowder concentration.^14^

In those studies, the SANS signal was assumed to result exclusively from the labeled chain form factor, assuming that any other contribution was removed by subtracting the scattering of the nearly contrast-matched crowder. However, in such concentrated systems, even mild polymer–solvent interactions contribute to the scattering. To account for such effects, the random phase approximation (RPA) formalism applies, which considers the contribution of the effective Flory–Huggins interaction parameter between the different components in a polymer mixture to the total structure factor. This theoretical framework is commonly invoked to analyze the structure^16^ and dynamics^17,18^ of polymer blends and, even though it works best for concentrated systems and melts, it has also been used to elucidate the structural and thermodynamical quantities of polymer solutions^19,20^ and gels.^21^ Here, we use a three-component RPA approach to analyze the SANS results on hydrogenated SCNPs in solutions of deuterated linear crowders and deuterated solvent. For this, we use PMMA-based SCNPs with 30% functional groups cross-linked with a trifunctional cross-linker. The crowder is deuterated PMMA in a solution of deuterated dimethylformamide (DMF). We compare the results of considering the RPA with the ones obtained under the abovementioned simplification.

## Materials and Methods

### Sample Preparation

**Scheme 1:**
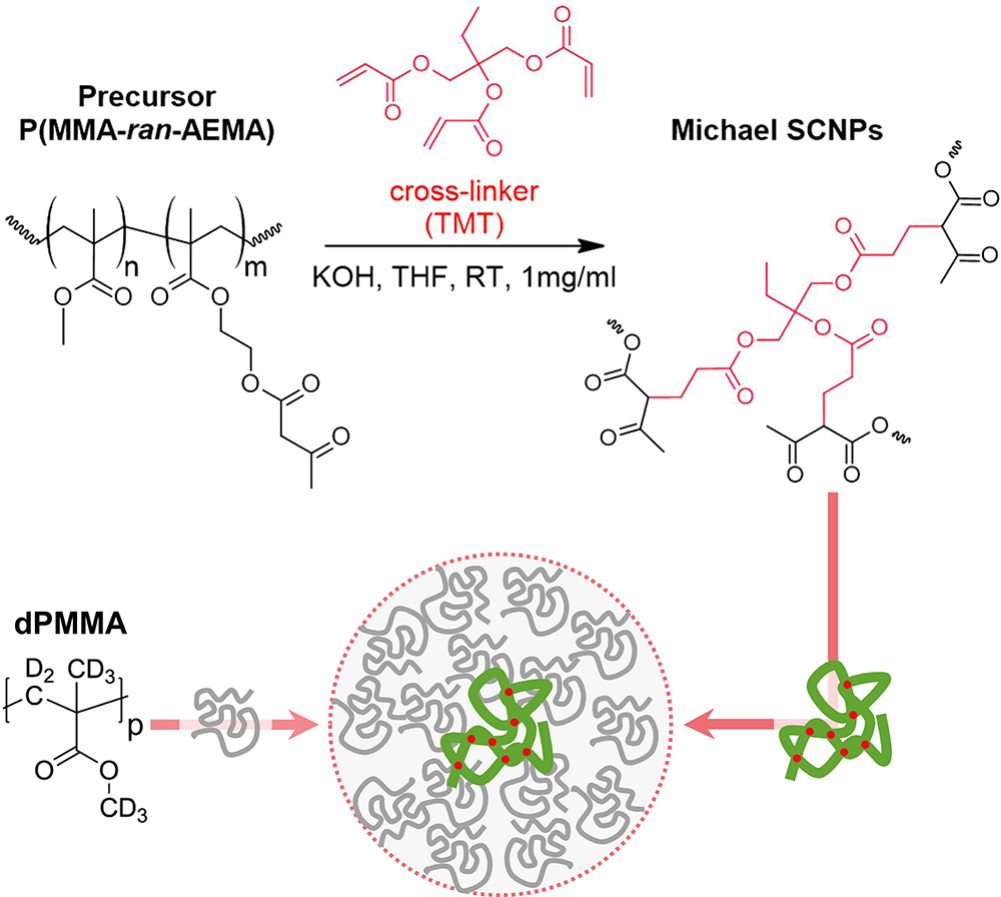
Synthesis of Poly(methyl methacrylate-*ran*-(2-acetoacetoxy)ethyl methacrylate) Single-Chain Nanoparticles. Schematic Illustration of the SCNPs under Crowding with dPMMA Chains in dDMF Is Included at the Bottom In the samples studied in this work, *n* = 0.69 and *m* = 0.31.

We investigate SCNPs and the corresponding linear precursors (Prec) as reference. The precursors consist of random copolymers of methyl methacrylate (MMA) and (2-acetoacetoxy)ethyl methacrylate (AEMA), namely, P(MMA_0.69_-*ran*-AEMA_0.31_), synthesized through reversible addition–fragmentation chain-transfer polymerization.^22^ The SCNPs were obtained through Michael addition of the trifunctional cross-linking agent trimethylolpropane triacrylate (TMT, 33 mol % to AEMA) (Sigma-Aldrich, technical grade) to β-ketoester functional groups of the precursors in a procedure described earlier^22^ (see Scheme 1). Two different molecular weights were investigated. Molecular weights and polydispersities of the samples (as determined by SEC/MALLS, see Supporting Information S.1) as well as other physicochemical parameters are displayed in Table 1.

**Table 1:** Molecular Characteristics of Precursors, SCNPs, Crowders, and the Solvent: Molecular Weight (*M*_w_) and Polydispersty Index (*M*_w_/*M*_n_), Mass Density (*d*), Scattering Length Density (ρ), Radius of Gyration (*R*_g_), Scaling Exponent (ν), and Overlap Concentration (c*)

		*M*_w_^a^ (kg/mol)	*M*_w_/*M*_n_^a^	*d* (g/cm^3^)	ρ (10^10^ cm^–2^)	*R*_g_^b^ (nm)	ν^b^	c*^c^ (mg/mL)
probes	Lo-Prec	33.1	1.12	1.21	1.27	5.3	0.481	46
	Lo-SCNPs	33.9	1.04			4.3	0.389	90
	Hi-Prec	247	1.35			16.2	0.556	12
	Hi-SCNPs	239.2	1.31			14.5	0.484	16
crowders	Lo-dPMMA	9.6	1.11	1.27	6.97	3.4	0.59	51
	Hi-dPMMA	99.1	1.09			10.9	0.59	16
solvent	dDMF			1.03	6.36			

a From SEC/MALLS in THF.

b From SANS in dilute solutions.

c .

The neutron scattering experiments were carried out on solutions with deuterated *N*,*N*-dimethylformamide (dDMF, 99.5 at. %, Acros Organics) as solvent. Crowded solutions were obtained by adding deuterated linear PMMA chains of two different molecular weights (dPMMA, Polymer Source, see Table 1). After synthesis and purification, stock solutions of precursors and SCNPs were prepared, and appropriate quantities of dPMMA were added immediately to reach the desired total concentration: in the case of low-*M*_w_ probes, *c*_Tot_ = *c*_probe_ + *c*_crowder_ = 20 + 180 mg/mL = 200 mg/mL, and in the high-*M*_w_ probes *c*_Tot_ = *c*_probe_ + *c*_crowder_ = 5 + 195 mg/mL = 200 mg/mL. The concentration of the probes is below the overlap concentration, estimated as  for the two cases (see Table 1). In the crowded samples, the total concentration of the polymer is above the overlap concentration of the probe. The neutron scattering length density ρ of dDMF is 6.36 × 10^10^ cm^–2^, close to that of dPMMA (see Table 1).

### SANS Measurements

SANS measurements were performed on the instrument KWS-2 at the Forschungs-Neutronenquelle Heinz Maier-Leibnitz (MLZ) in Garching, Germany.^23^ Measurements were carried out at room temperature using a neutron wavelength of λ = 5 Å. For the low-molecular-weight probes, two sample–detector distances were used (2 and 8 m) with 8 m collimation. For the bigger macromolecules, additional measurements were conducted at a distance of 19.9 m with 20 m collimation. Data from different detector positions were merged applying the same calibration factor for all the samples. Samples were contained in 2 mm thick quartz cuvettes (QS, Hellma). The sensitivity of the detector elements was accounted for by comparing to the scattering of a 1.0 mm sample of water, and a Plexiglas measurement was used for absolute scaling. Sample thickness, transmission, detector dead time, and electronic background were considered, and the background due to the scattering of the cell filled with deuterated solvent was subtracted from the sample measurements with H-labeled macromolecules for the RPA analysis. Finally, the azimuthally averaged scattered intensities were obtained as a function of the wave-vector magnitude, *Q* = 4π sin(θ/2)/λ, where θ is the scattering angle. In the data analyses, the additional incoherent background arising mainly from the hydrogens in the polymers was fitted as a constant *I*_inc_ at high-*Q* and subtracted from the data such that *I*(*Q*) = *I*_exp_(*Q*) – *I*_inc_, where *I*_exp_ is the experimentally obtained data (see the Supporting Information for further details). We note that the influence of the background in our case is not that critical due to the high coherent scattering intensity of the samples compared to the incoherent background and, not less important, due to the wide *Q*-range of the slope between π/*R*_g_ and the point where the curve flattens. This is even less important for the high-molecular-weight probes where *I*(0) is higher and π/*R*_g_ is lower. The error bars for *Q* < 0.3 Å^–1^ are smaller than the size of the points (see Figure S4).

### SANS Analysis

For a binary system, such as a polymer in solution, the total measured differential coherent scattering cross section per unit sample volume, *I*(*Q*), depends on the contrast between the two components as
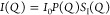
1where *I*_0_ is the forward scattering (*Q* → 0 value), *P*(*Q*) is the form factor, and *S*_I_(*Q*) is the structure factor accounting for intermolecular interactions between particles. The forward scattering can be written as *I*_0_ = ϕΔρ^2^*V*, where ϕ is the volume fraction of scatterers, Δρ is the scattering contrast given by the difference in scattering length density ρ between the components, *V* = *M*_w_/*dN*_A_ is the volume of the scatterer; with *M*_w_ being the weight-average molecular weight, *d* the mass density, and *N*_A_ the Avogadro number. Note that eq 1 assumes monodisperse objects. Under high dilution conditions, interactions between different macromolecules are negligible and the associated structure factor can be considered close to unity, *S*_I_(*Q*) ≈ 1. Thus, the *Q*-dependence of the measured curve is determined just by the form factor of the particles in solution, *P*(*Q*).

For linear polymers and SCNPs in dilute solutions,^24^ to take into account molecular conformation including excluded volume effects, a generalized Gaussian coil form factor *P*(*Q*, *R*_g_, ν)^25^ should be used
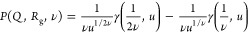
2awith
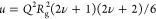
2b
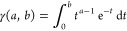
2cwhere *R*_g_ is the radius of gyration and ν is the scaling exponent. Fully swollen linear chains have a ν exponent of 3/5 (good solvent), Gaussian chains of 1/2 (linear chains in θ-solvent), and collapsed macromolecules of 1/3 (bad solvent). This form factor is normalized to 1 at low *Q*. The Debye function is recovered from eq 2 for ν = 0.5. When the solvent–polymer interactions are strong or in the semidilute and concentrated regime where intermolecular interactions are non-negligible, the scattered intensity is affected and formalisms such as the RPA must be used to account for such effects.

### Random Phase Approximation for a Three-Component System

The RPA theory has been used to describe the SANS data. In the following, we present the scattering function of a polymer solution consisting of a mixture of protonated and deuterated polymers in this framework. For incompressible mixtures,^26−28^ the macroscopic scattering cross section is given by
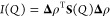
3where, for a three-component system, **S**(*Q*) is a 2 × 2-matrix and **Δ**ρ is a 2-column vector for the scattering length density differences relative to the background (third component) and **Δ**ρ^T^ is its transpose. The inverse structure matrix for this system can be written as
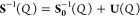
4**S**_0_(*Q*) is a matrix of noninteracting (bare) structure factors, which for homopolymer solutions is diagonal. The excluded volume interactions are contained in matrix **U**, which can be expressed in terms of the bare structure factor for the background component *S*_33_^0^(*Q*) and the Flory–Huggins interaction parameters χ_*ij*_
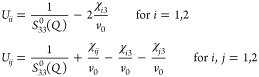
5where *v*_0_ is a reference volume. Thus, for a three-component system consisting of two polymers and a solvent, with component 1 being the probe polymer, component 2 the crowder, and component 3 the solvent, the scattered intensity is given as the summation of squares of scattering length density differences between polymer chains and solvent molecules, multiplied by the structure factors
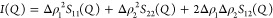
6

According to eq 4, the fully interacting system structure factors can be written as
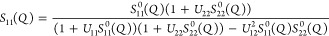
7a
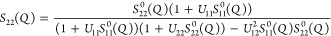
7b
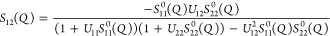
7c

The single-chain form factors for homopolymers can be expressed as
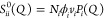
8where *N*_*i*_ is the degree of polymerization for the component *i*, ϕ_*i*_ its volume fraction, *v*_*i*_ the molar volume of a segment of the chain, and *P*_*i*_(*Q*) its form factor. For the solvent, *N*_3_ = 1, *P*_3_(*Q*) = 1, and thus, the bare structure factor of component 3 can be written as *S*_33_^0^ = *v*_3_(1 – ϕ_p_), with ϕ_p_ = ϕ_1_ + ϕ_2_ the total volume fraction of polymer in the solution.

Equation 7 can be simplified under the assumptions that the probe and the crowder are compatible (χ_12_ = 0). In our systems, this assumption is made because, by experimental design, the precursors and SCNPs have a chemical composition very similar to the crowder. In addition, the interaction between both polymers and the solvent is assumed to be the same (χ_13_ = χ_23_ = χ), thus
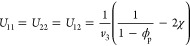
9where we have chosen *v*_3_ as the reference volume.

In principle, the RPA was derived for polymers that exhibit Gaussian statistics, where *P*(*Q*) is described by the Debye scattering function, *i.e.*, the conformation linear polymers adopt in θ-solvent conditions and in bulk. However, in polymers in other solvent conditions or macromolecular conformations leading to a different scaling exponent, a generalized Gaussian coil form factor (eq 2) has to be used. This form factor yielded reasonable results for the analysis of SANS experiments in polymer solutions using the RPA approach.^19,20^

Equation 6 could be further simplified under contrast-matching conditions between the crowder and the solvent (Δρ_2_ = 0), leading to
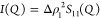
10We want to emphasize that contrast-matching is different to zero-average contrast (ZAC) conditions, fulfilled for a system where the two polymers have the same degree of polymerization *N*_1_ = *N*_2_, equal form factor *P*_1_(*Q*) = *P*_2_(*Q*), and the same thermodynamic properties with respect to the solvent, *i.e.*, the quality of the solvent is the same for both polymers χ_13_ = χ_23_ = χ, the following condition is satisfied
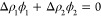
11and the interaction between both polymers is zero (χ_12_ = 0). Under ZAC conditions, the equation  is obtained,^29^ and the single-chain form factor, which contains information on the intramolecular correlations, is accessible.

Lastly, if the Flory–Huggins interaction parameter fulfills the condition
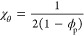
12the excluded volume interactions are zero (*U*_11_ = *U*_22_ = *U*_12_ = 0) and the system is under θ-solvent conditions. Under these circumstances, the fully interacting system structure factors *S*_11_(*Q*) and *S*_22_(*Q*) are equal to the corresponding single-chain form factors (eq 8), while *S*_12_(*Q*) = 0 (see eq 7). Under that condition, eq 6 can be written as
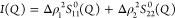
13If the crowder is contrast-matched with the solvent, Δρ_2_ = 0 and eq 1 is recovered. Equation 12 marks the threshold between good solvent and poor solvent conditions: if χ is lower than , the excluded volume interaction parameter *U* is positive (see eq 9) and the system is in good solvent conditions. On the contrary, if , *U* is negative and the system is in poor solvent conditions.

## Results and Discussion

To study the effect of the crowding environment on the structure of both precursors and their SCNPs, we used SANS, where the scattering intensity of the protonated precursors and SCNPs is highlighted due to the low contrast between the deuterated crowders and the deuterated solvent (Table 1). We considered two different molecular weights for the protonated precursors; low-*M*_w_ = 33.1 kDa and high-*M*_w_ = 247 kDa. For both macromolecules, we kept their concentration in all the solutions below their own overlap concentration c*, *i.e.*, 20 mg/mL for low-*M*_w_ probes and 5 mg/mL for high-*M*_w_ probes. In the crowded samples, the total concentration was kept at 200 mg/mL. Crowded conditions were induced using crowders with two molecular weights.

**Figure 1:**
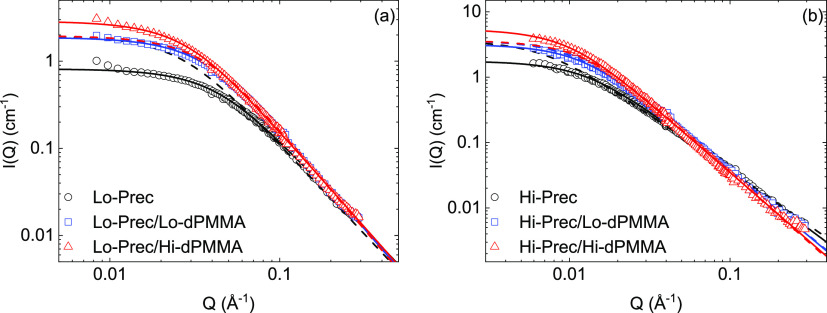
Scattered intensities *I*(*Q*) of (a) low-molecular-weight and (b) high-molecular-weight precursors in dilute (black circles) and under crowding conditions with low-molecular-weight crowders (blue squares) and high-molecular-weight crowders (red triangles). Lines are fits using eq 1 with a generalized random coil form factor (eq 2) with forward scattering *I*_0_ for *Q* = 0 as a fitting parameter (solid lines), and with the forward scattering fixed to that defined by the scattering length density and the molecular parameters, as in eq 1 (dashed lines).

To illustrate the need for using the RPA approach in these cases, we consider the SANS curves of the linear precursors in dilute solutions and in the presence of crowders. Figure 1 shows the SANS results obtained for the low- and high-molecular-weight precursors in the dilute regime and under crowding conditions. In the case of the concentrated solutions, the contribution of the crowders to the scattered intensity has been taken into account by subtracting the signal from solutions of the deuterated crowder in the deuterated solvent as it was considered in the previous work^14^ (see the Supporting Information for details on background subtraction). In all cases, the shape of the scattering curves corresponds to that of a polymer coil with a Guinier region at low-*Q* and a *Q*^–1/ν^ power law at intermediate-*Q* related to the chain fractal dimension. In both systems, upon crowding, there is an increase in the slope, which is indicative of more compact objects. In addition, it can be seen that the forward scattering intensity increases under crowding conditions. Generally, an increase in forward scattering in binary polymer/solvent systems can be due either to molecular weight increase, caused by chemical intermolecular cross-linking of unreacted functional groups, or due to aggregation, caused by attractive physical interactions between the polymers or bad solvent conditions.

Recently, our group studied aggregation effects in PS-based SCNPs under crowding in molecular-weight symmetric and asymmetric systems.^15^ There, it was observed that low-molecular-weight crowders added to high-*M*_w_ SCNPs solutions produce depletion interactions, destabilizing the suspension and causing chemical aggregation mediated by unbound reactive groups. This is not the case for the samples investigated in this work since the same intensity increase at low-*Q* values can be observed when adding crowders to the solutions of both, precursors and SCNPs (see Figures S2 and S3), irrespective of the presence of reactive groups, and it happens in both symmetric and asymmetric polymer mixtures. Therefore, the observed changes in forward scattering do not appear to be related to aggregation.

Furthermore, according to eq 1, in the absence of interactions, *i.e.*, *S*_I_(*Q*) ≃ 1, the value of the forward intensity should be given by the molecular volume as well as its concentration and contrast (see Table 1). However, when using eq 1 to describe the data (Figure 1, solid lines), in the case of dilute samples without any crowder, the forward scattering value *I*_0_ given by the fitting is lower than that determined by the molecular parameters of the studied systems, Δρ^2^ϕ*V* (see Figure 1, dashed lines). The fitting parameters are given in Table S2. This mismatch in the intensity at low-*Q* values in the dilute samples without crowding can be attributed to repulsive interactions between the probe chains. In fact, it was observed in a previous study^30^ that, at the concentration of 20 mg/mL used for the low-molecular-weight precursor and SCNP system, the SCNPs in solution display non-negligible intermolecular interactions leading to a structure factor which deviates from unity. When crowders are added to the solutions, these repulsive interactions are screened due to the presence of the dPMMA chains, leading to an increase in the forward scattering intensity value. We note that the high-molecular-weight crowder leads to a higher intensity increase. In addition, the forward scattering does not correlate with the molecular weight asymmetry in the polymer mixture, ruling out depletion interactions. Therefore, polymer–solvent interactions must be considered in the overall structure factor.

The scattered intensity can be described following the RPA approach for three components (probe, crowder, and solvent) using eqs 6, 7, and 8. In this case, note that the background subtracted corresponds to the deuterated solvent (without crowders) and the incoherent contribution (see Supporting Information Section S.4). Assuming χ_12_ = 0 and χ_13_ = χ_23_ = χ (see eq 9), there are a total of 5 variable parameters: the radii of gyration (*R*_g,1_, *R*_g,2_) and the scaling exponents (ν_1_, ν_2_) of both polymers, and the Flory–Huggins interaction parameter (χ). The rest of the parameters can be fixed according to the sample composition and molecular parameters listed in Table 2. Here, we consider the reference unit for our precursors or SCNPs what we call “effective” monomer, whose properties are the result of averaging over the copolymer components. Thus, the molar mass *m*_0_ of the effective monomer is obtained as 0.69 × *m*_0,MMA_ + 0.31 × *m*_0,AEMA_ = 135.6 g/mol. From this value, we obtained the degree of polymerization and molar volume of the probes.

**Table 2:** Fixed Parameters for RPA Curve Fitting Using Eqs 6, 7 and 8: Degree of Polymerization (*N*_*i*_), Volume Fraction (ϕ_*i*_), and Molar Volume (*v*_*i*_)

	probe		crowder		solvent
degree of polymerization	low-*M*_w_	*N*_1_ = 244	Lo-dPMMA	*N*_2_ = 90	*N*_3_ = 1
	high-*M*_w_	*N*_1_ = 1821	Hi-dPMMA	*N*_2_ = 920	
polymer volume fraction	low-*M*_w_	ϕ_1_ = 0.017		ϕ_2_ = 0.141	ϕ_3_ = 1–ϕ_p_^a^
	high-*M*_w_	ϕ_1_ = 0.004		ϕ_2_ = 0.154	=0.842
molar volume		*v*_1_ = 187 Å^3^		*v*_2_ = 141 Å^3^	*v*_3_ = 129 Å^3^

a ϕ_p_ = ϕ_1_ + ϕ_2_ is the total volume fraction of polymer in the solution.

As the single-chain form factors in eq 7 are strongly correlated, it is hard to treat the radii of gyration and the scaling exponents as independent fitting parameters.^31^ Thus, the values of *R*_g_ and ν of the crowders were estimated and fixed during the fitting procedure, leaving only three free fitting parameters in the model. The estimation of *R*_g_ and ν for the crowders was made in the following way: first, from SAXS and SANS measurements, these parameters were determined in dilute conditions; from them, the values corresponding to high concentration were estimated according to computer simulations performed on linear polymers in the semidilute regime^10^ (see the Supporting Information). These values, along with the final values of the fit parameters for SCNPs and precursors, are given in Table 3 for a better comparison.

**Table 3:** Fitting Parameters for RPA Curve Fitting Using Eqs 6, 7 and 8

		precursors			SCNPs			crowders^a^	
		*R*_g_ (nm ±0.1)	ν (±0.005)	χ (±0.002)	*R*_g_ (nm ±0.1)	ν (±0.005)	χ (±0.002)	*R*_g_ (nm)	ν
low-*M*_w_	dilute	5.3	0.481	0.391	4.3	0.389	0.405		
	with Lo-dPMMA	4.4	0.433	0.545	4.4	0.404	0.450	3.1	0.550
	with Hi-dPMMA	4.3	0.395	0.596	4.3	0.375	0.595	9.3	0.530
high-*M*_w_	dilute	16.2	0.556	0.462	14.5	0.484	0.428		
	with Lo-dPMMA	14.1	0.527	0.590	12.5	0.480	0.546	3.1	0.550
	with Hi-dPMMA	14.5	0.512	0.597	11.4	0.431	0.594	9.3	0.530

a Fixed according to SAXS/SANS measurements and computer simulations.^10^

**Figure 2:**
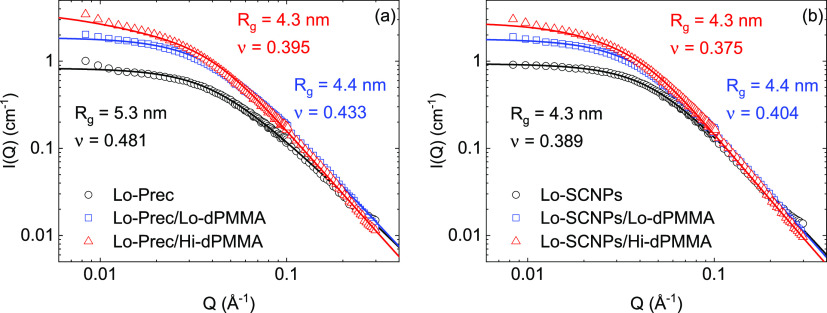
Scattered intensities *I*(*Q*) of low-molecular-weight (a) precursors and (b) SCNPs in dilute (*c*_Tot_ = 20 mg/mL, black circles) and under crowding conditions (*c*_Tot_ = *c*_probe_ + *c*_crowder_ = 20 + 180 mg/mL) with a low-molecular-weight crowder (blue squares) and high-molecular-weight crowder (red triangles). Solid lines are RPA fits with parameters given in the legends and in Table 3.

**Figure 3:**
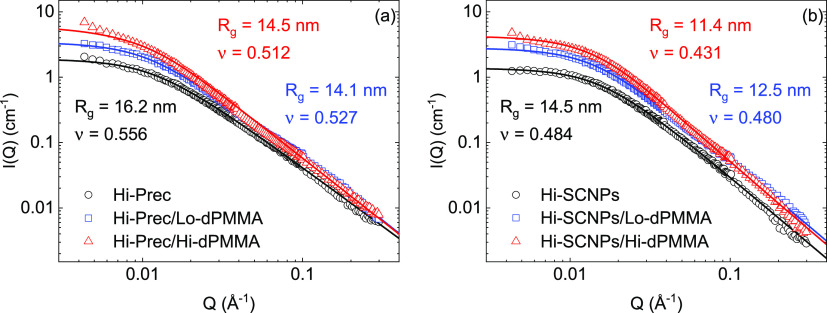
Scattered intensities *I*(*Q*) of high-molecular-weight (a) precursors and (b) SCNPs in dilute (*c*_Tot_ = 5 mg/mL, blue circles) and under crowding conditions (*c*_Tot_ = *c*_probe_ + *c*_crowder_ = 5 + 195 mg/mL) with a low-molecular-weight crowder (blue squares) and a high-molecular-weight crowder (red triangles). Solid lines are RPA fits with parameters given in the legends and in Table 3.

Figure 2 displays the scattered intensities obtained from SANS measurements for the low-*M*_w_ system along with the description obtained using the RPA formalism described above. The equivalent graphs for the high-molecular-weight precursor and SCNPs are shown in Figure 3. The fits describe the experimental results equally well as letting the forward scattering free and neglecting any other contributions, yet here the molecular weights, concentrations, and scattering length densities are fixed and the only free parameters are *R*_g_, ν and χ, which are listed in Table 3.

**Figure 4:**
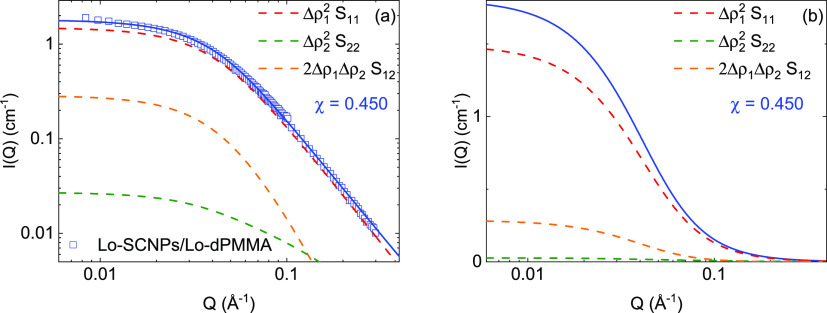
Scattered intensities *I*(*Q*) of low-molecular-weight SCNPs in crowding with Lo-dPMMA with RPA fits (solid lines). Each contribution on eq 6 is represented in a different color (dashed lines, see legend). Data in (a) are represented in logarithmic scale. In (b), the fitting function together with the components is shown in linear scale.

**Figure 5:**
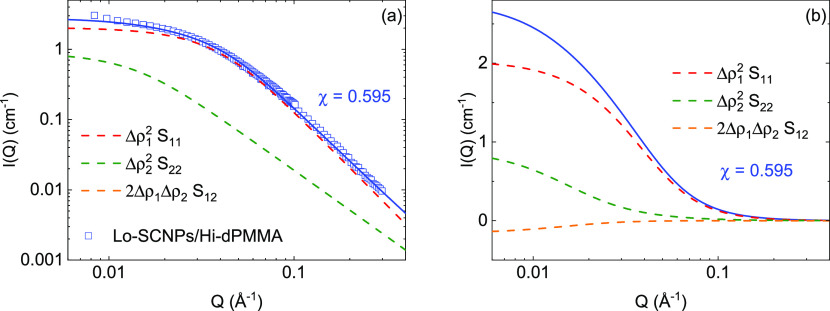
Scattered intensities *I*(*Q*) of low-molecular-weight SCNPs in crowding with Hi-dPMMA with RPA fits (solid lines). Each contribution on eq 6 is represented in a different color (dashed lines, see legend). Data in (a) are represented in logarithmic scale. In (b), the fitting function together with the components is shown in linear scale.

To visualize the contribution of each of the terms in eq 7 to the total scattered intensity described by the three-component RPA formalism (eq 6), we have selected the case of the Lo-SCNPs in crowding conditions with low- and high-molecular-weight dPMMA. The contribution of each one of the components in eq 7 multiplied by the corresponding contrast factor is represented in Figure 4 for the Lo-SCNPs with the Lo-dPMMA crowder and in Figure 5 for the Lo-SCNPs with the Hi-dPMMA crowder. If the interaction parameter χ is lower than , the excluded volume interaction parameter *U* is positive and the system is in good solvent conditions. Also, *S*_12_ is negative and the third (cross) term in eq 6 is positive (Δρ_1_ < 1). This is the case represented in Figure 4, where χ = 0.450 < χ_θ_. The cross-term 2Δρ_1_Δρ_2_*S*_12_ contributes more than the second term in eq 6, which reflects the contrast between the crowder and the solvent. On the contrary, when , the interaction parameter *U* is negative, meaning that the self-attractions become important and the polymer becomes less soluble. In that case, 2Δρ_1_Δρ_2_*S*_12_ is negative. That is the case represented in Figure 5, where χ = 0.595 > χ_θ_, and the term Δρ_2_^2^*S*_22_ contributes substantially to the overall curve.

Thus, in our systems, even though the scattering length density of the crowder is very close to that of the solvent and Δρ_1_ is much larger than Δρ_2_, the contributions of the crowder scattering as well as the cross-term are non-negligible, and they are more relevant in the samples crowded with high-molecular-weight dPMMA. We note that neglecting the *S*_22_ and the cross-term contributions would not describe the observed intensities.

Analyzing the probe form factor parameters (*R*_g_ and ν) obtained from the RPA fits, we make several observations. First, for both molecular weights, the SCNP formation *via* intramolecular cross-linking leads to a reduction of the radius of gyration as well as the scaling exponent. The relative size reduction upon SCNP formation is more pronounced in the case of the higher-molecular-weight system, as expected from theory and observed experimentally.^32,33^ On the other hand, for the crowder concentration here investigated, the presence of the crowder produces a significant size reduction on the high-molecular-weight probes, while it has only a very small effect on the low-molecular-weight probes. These findings are qualitatively in agreement with the conclusions from the previous study,^14^ where the overlap concentration of the SCNP was identified as the crossover concentration above which the macromolecule starts shrinking.

The interaction parameter χ varies with sample composition (see Table 3). In principle, the Flory–Huggins parameter is related to the solvent quality. Lower values than the threshold value given in eq 12 indicate good solvent conditions and higher values, poor solvency. In the dilute regime (ϕ_p_ ≪ 1), the threshold is at χ_θ_ = 0.5. Our results show that in the dilute regime, precursors and SCNPs are in good solvent conditions. However, for the samples in crowded conditions, the value of the interaction parameter is close to the threshold value (at the concentration here investigated, χ_θ_ ≈ 0.594). In particular, when crowding is induced with Hi-dPMMA, χ > χ_θ_. These results suggest that for PMMA in semidilute solutions, DMF is a worse solvent. We note that the values found here are close to the ones reported for PMMA in DMF semidilute solutions χ_PMMA/DMF_ ∼ 0.56.^34^

## Conclusions

The conformation of PMMA-based SCNPs and their corresponding precursors in dilute and under crowding conditions with linear PMMA chains has been investigated by SANS varying the molecular weight of the probes and the crowders. In spite of using deuterated crowders in deuterated solvent, the forward scattering intensity increases in crowding conditions, with the high-molecular-weight crowder leading to a higher increase. Thus, the scattered intensity was analyzed in terms of an RPA model for the three components (probe, crowder, and solvent) to consider the polymer–solvent interactions and cross-correlations.

The form factor parameters *R*_g_ and ν of the precursors and the SCNPs, as well as the Flory–Huggins interaction parameter between the PMMA and the solvent, were obtained in dilute and crowded conditions. The presence of the crowder produces a size reduction on the probes. For the crowder concentration here investigated, the size reduction is more pronounced in the high-molecular-weight probes. Applying the three-component RPA model leads to a more accurate estimation of the form factor parameters since it considers all the contributions to the scattering. Corrections of about 30% in the value of *R*_g_ and about 10% in the scaling exponent ν are obtained with respect to the values estimated neglecting interactions and cross-correlations. We note that even considering these corrections, the trends reported in previous works on similar systems are reproduced. The RPA model allows the determination of the Flory–Huggins interaction parameter, which varies with the sample composition, indicating that in the dilute regime, DMF is a good solvent for PMMA while in crowded conditions, the polymer becomes less soluble.

Studies on SCNP structure in crowded media as that here presented can be considered as a basis for understanding the structural conformation of important and ubiquitous biomacromolecules as intrinsically disorder proteins and unfolded coils in dense environments—the natural state *in vivo* conditions.

## Supporting Information Available

Experimental details of the SEC/MALLS measurements; SAXS/SANS characterization of the PMMA/dPMMA crowders; analysis of the SANS data in terms of the generalized Gaussian coil; and details on background subtraction (PDF)
