# Supplementary material for: Structure of Single-Chain Nanoparticles under Crowding Conditions: A Random Phase Approximation Approach
Source: Macromolecules. 2023 Nov 3;56(21):8971–9. doi: 10.1021/acs.macromol.3c01333 (PMC10654932; doi:10.1021/acs.macromol.3c01333)
Supplement: Supplementary file 1 — ma3c01333_si_001.pdf [file ma3c01333_si_001.pdf]

Supporting Information: Structure of Single-Chain Nanoparticles Under Crowding Conditions: A Random Phase Approximation Approach

Beatriz Robles-Hernández,[*,†] Marina González-Burgos,[‡] Paula Malo de Molina,[*,‡,¶] Isabel Asenjo-Sanz,[‡] Aurel Radulescu,[§] José A. Pomposo,[‡,¶,∥] Arantxa Arbe,[‡] and Juan Colmenero[‡,∥,†]

†*Donostia International Physics Center (DIPC), 20018 Donostia-San Sebastián, Spain*

‡*Centro de Física de Materiales/Materials Physics Center (CFM/MPC), 20018 Donostia-San Sebastián, Spain*

¶*IKERBASQUE – Basque Foundation for Science, 48009 Bilbao, Spain*

§*Forschungszentrum Jülich GmbH, Jülich Centre for Neutron Science (JCNS) at Heinz Maier-Leibnitz Zentrum (MLZ), 85748 Garching, Germany*

∥*Department of Polymers and Advanced Materials: Physics, Chemistry and Technology, University of the Basque Country UPV/EHU, 20018 Donostia-San Sebastián, Spain*

E-mail: beatriz.robles@ehu.eus; p.malodemolina@ehu.eus

S.1 Size-Exclusion Chromatography / Multi-Angle Laser Light Scattering (SEC/MALLS) Measurements

SEC/MALLS measurements were performed at 30 °C on an Agilent 1200 system equipped with Agilent PLgel 10 μm Guard and PLgel 10 μm MIXED-B LS columns, a differential refractive index (dRI) detector (Optilab Rex, Wyatt) and a multi-angle laser light scattering (MALLS) detector (MiniDawn Treos, Wyatt). Data analysis was performed with ASTRA Software from Wyatt (PMMA-based polymers: $\mathrm{d}n/\mathrm{d}c = 0.083$). THF was used as eluent at a flow rate of 1 mL/min and the samples were injected at a concentration of 4 mg/mL.

S.2 Characterization of the dPMMA crowders in dilute solutions

Small Angle X-Ray Scattering (SAXS) experiments on dilute solutions of deuterated PMMA crowders in deuterated DMF (low-molecular weight, 1 mg/mL; high-molecular weight, 5 mg/mL) were performed to determine the radius of gyration, R_g and the scaling exponent, ν. Measurements were carried out at 25 °C on a Rigaku 3-pinhole PSAXS-L instrument operating at 45 kV and 0.88 mA. The MicroMax-002 + X-ray Generator Systems is composed by a microfocus sealed tube source module and an integrated X-ray generator unit that produces Cu- Kα transition photons of wavelength $\lambda = 1.54$ Å. The flight path and the sample chamber were under vacuum. The scattered X-rays were detected on a two-dimensional multi-wire X-ray detector (Gabriel design, 2D-2000$\times$). This gas-filled proportional type detector offers a 200 mm diameter active area with ca. 200 micron resolution. The azymuthally averaged scattered intensities were obtained as a function of scattering vector. Reciprocal space calibration was done using silver behenate as standard. The solutions were filling boron-rich capillaries with an outside diameter of 2 mm and a wall thickness of about 0.01 mm. The sample was placed in transmission geometry, with a sample to detector distance

of 2 m, covering a Q-range between about 0.01 and 0.2 Å^{-1}. Each sample was measured for 1 h. The solvent was measured under the same conditions and properly subtracted from measurements on the solution.

The scattered intensity along with the fitting to Eqs. 1 and 2 are shown in Fig. S1. The corresponding fitting parameters are reported in Table S1. For the Lo-PMMA sample, the value obtained for the scaling exponent is 0.59, as expected for linear chains in good solvent conditions, with a value of R_g = 3.4 nm (see Table S1). For the high-molecular weight dPMMA in dilute conditions, from the SAXS measurements it was only possible to obtain the scaling exponent, with a value of 0.59. SANS measurements of the Hi-dPMMA crowder on protonated DMF were performed to determine the value of radius of gyration, and to confirm the value of the scaling exponent obtained from SAXS experiments (see Table S1).

To estimate the values of the radius of gyration and the scaling exponent at 200 mg/mL, large-scale computer simulations results[S1] were used. They predict a reduction of the radius of gyration of about an 8% and a value for the scaling exponent of 0.55, for the Lo-dPMMA, and a reduction of R_g of about a 15% and a value of $\nu = 0.53$, for the Hi-dPMMA.

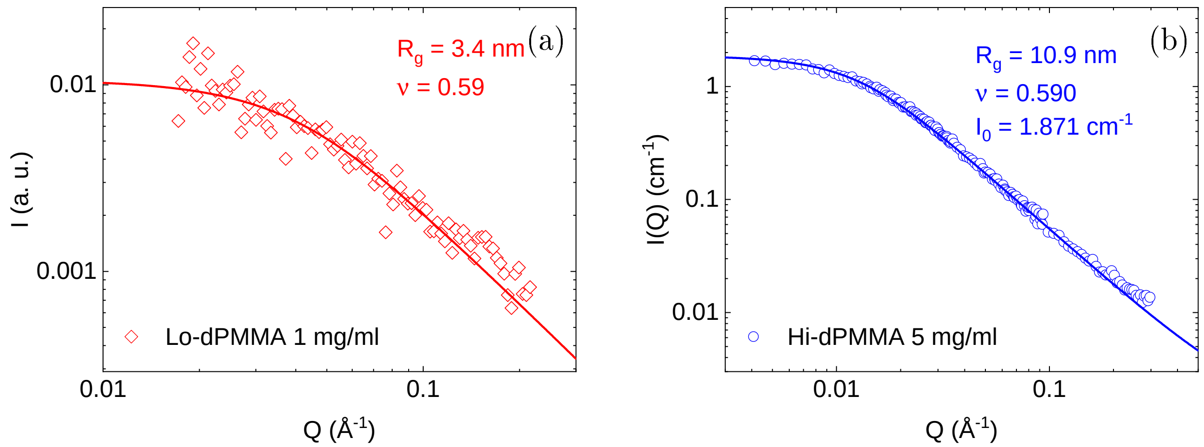

Figure S1: Scattered intensity $I(Q)$ of (a) Lo-dPMMA crowder at 1 mg/mL in deuterated DMF, obtained from SAXS measurements and (b) Hi-dPMMA crowder at 5 mg/mL in protonated DMF, obtained from SANS experiments. Lines are the fitting to Eq. 1 using a generalized Gaussian coil model as form factor, see Eq. 2. The obtained parameters are shown in the legend.

Table S1: Fitting parameters obtained for deuterated crowders in dilute solutions of DMF in terms of generalized Gaussian coil form factor without interactions (Eqs. 1 and 2) with the forward scattering I_0 as a fitting parameter.

	R_g (nm)	ν	I_0 (cm^{-1})
Lo-dPMMA	3.4^a	0.590^a	
Hi-dPMMA	10.9^b	0.590^b	1.871^b

[a] from SAXS at 1 mg/mL in dDMF
[b] from SANS at 5 mg/mL in hDMF

S.3 Analysis in terms of a generalized Gaussian coil form factor without interactions

For comparison, all the samples were also analyzed in terms of a generalized Gaussian coil without interactions (Eqs. 1 and 2). Under some conditions, Eq. 6 can be reduced to Eq. 1: when the Flory-Huggins interaction parameter $\chi = \frac{1}{2(1-\phi_\mathrm{p})}$, the excluded volume interactions vanish ($U_{11} = U_{22} = U_{12} = 0$) and the fully interacting system structure factors $S_{11}(Q)$ and $S_{22}(Q)$ are equal to the corresponding single-chain form factors while $S_{12}(Q) = 0$ (see Eq. 7), leading to

$$I(Q) = \Delta\rho_1^2 S_{11}^0(Q) + \Delta\rho_2^2 S_{22}^0(Q). \tag{S.1}$$

Thus, to perform this analysis, the cell filled with deuterated solvent and deuterated crowder was subtracted from the sample measurements with protonated molecules, and the resulting data were fitted using Eqs. 1 and 2 with the forward scattering I_0 as a fitting parameter.

The scattered intensities of the low-molecular weight and the high-molecular weight probes in dilute and under crowding conditions with the corresponding fitting curves are represented in Figs. S2 and S3, respectively. The values obtained for the fitting parameters are reported in Table S2.

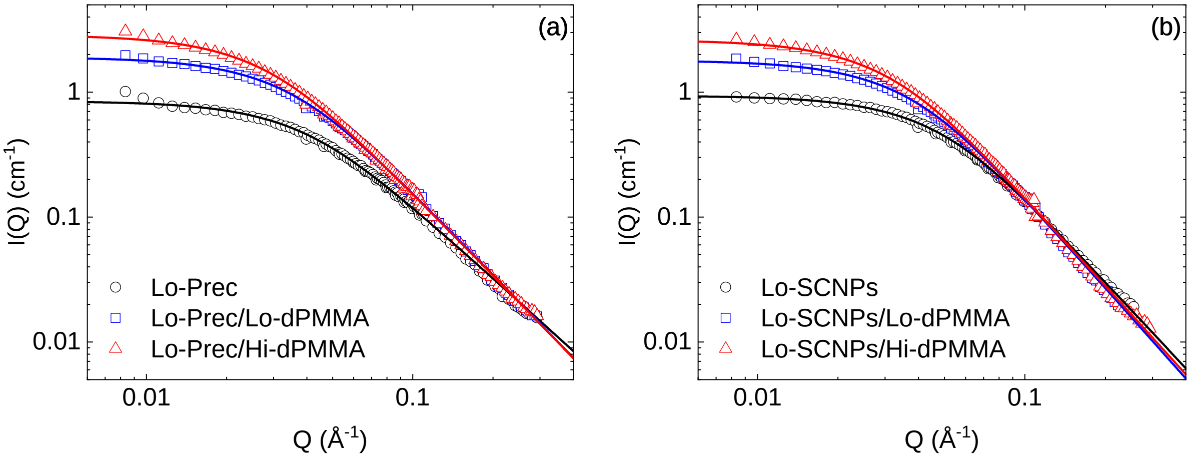

Figure S2: Scattered intensities $I(Q)$ of low-molecular weight (a) precursors and (b) SCNPs in dilute (c_{Tot} = 20 mg/mL, black circles) and under crowding conditions ($c_{\mathrm{Tot}} = c_{\mathrm{probe}} + c_{\mathrm{crowder}}$ = 20 mg/mL + 180 mg/mL) with Lo-dPMMA (blue squares) and Hi-dPMMA (red triangles) with generalized Gaussian coil fits (solid lines).

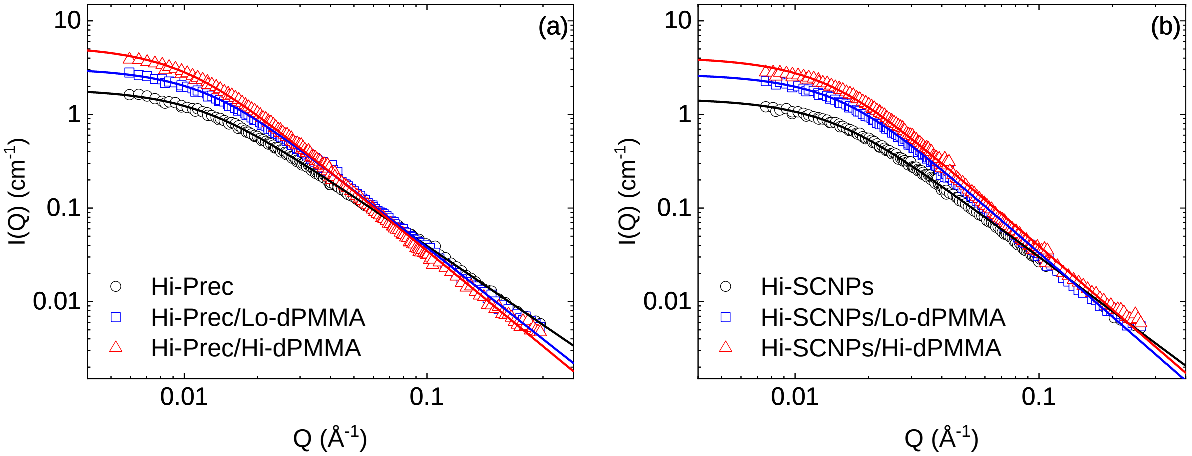

Figure S3: Scattered intensities $I(Q)$ of high-molecular weight (a) precursors and (b) SCNPs in dilute (c_{Tot} = 5 mg/mL, black circles) and under crowding conditions ($c_{\mathrm{Tot}} = c_{\mathrm{probe}} + c_{\mathrm{crowder}}$ = 5 mg/ml + 195 mg/mL) with Lo-dPMMA (blue squares) and Hi-dPMMA (red triangles), with generalized Gaussian coil fits (solid lines).

Table S2: Fitting parameters for generalized Gaussian coil form factor without interactions curve fitting using Eqs. 1 and 2.

		precursors			SCNPs		
		R_g (nm)	ν	I_0 (cm^{-1})	R_g (nm)	ν	I_0 (cm^{-1})
low-M_w	dilute	3.6	0.495	0.838	3.3	0.437	0.947
	with Lo-dPMMA	4.5	0.452	1.901	4.2	0.415	1.795
	with Hi-dPMMA	5.5	0.455	2.869	5.1	0.423	2.619
high-M_w	dilute	11.9	0.564	1.900	9.0	0.482	1.300
	with Lo-dPMMA	12.3	0.487	3.144	10.2	0.440	2.723
	with Hi-dPMMA	15.3	0.468	5.453	11.5	0.443	4.118

S.4 Background subtraction for RPA analysis

To perform the RPA analysis, first the cell with the solvent (dDMF) was subtracted from the data. Then, the additional incoherent background arising mainly from the hydrogens in the polymers was fitted at high-Q as a constant, $I_{\rm inc}$, and subtracted from the data such that the curves shown in this work are $I(Q) = I_{\rm exp}(Q) - I_{\rm inc}$, where $I_{\rm exp}$ is the experimentally obtained data. Figure S4 shall serve to clarify this procedure: in the inset are represented the measurements of the sample Lo-Prec/Lo-dPMMA solution (black circles) and of the solvent dDMF (red circles), and in the main figure the black squares correspond to the subtraction of these curves. The green line is the performed RPA fit, and the red line is the constant background obtained from the high-Q fit, $I_{\rm inc}$. The curves shown in this work are the blue squares, which correspond to the black squares minus the red line, and the black line, which corresponds to the green line minus the red line. Fig. S5 shows in the Porod representation that the fit with the background is very good and yields the same value for $I_{\rm inc}$.

Fig. S6 (panel a) shows the Kratky representation of the same data as in Fig. S4 as well as the equivalent data corresponding to the Lo-Prec/Hi-dPMMA sample (panel b). The data with background subtraction in Fig. S6 shows a negative slope of high-Q for both

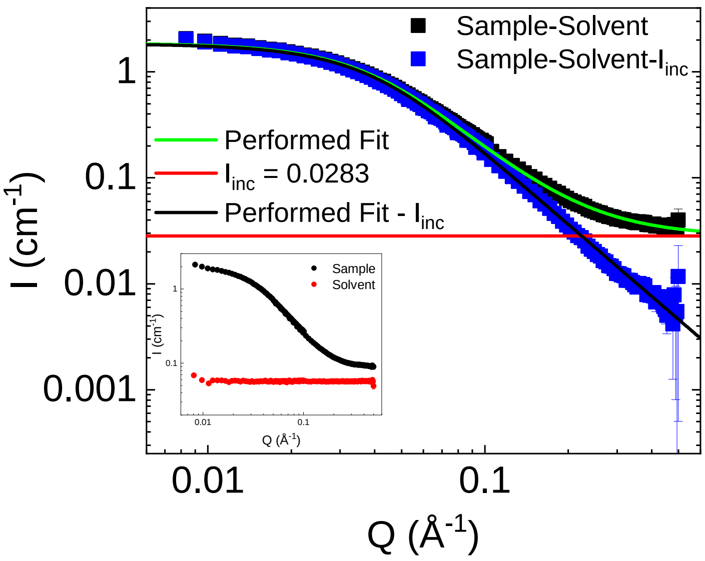

Figure S4: Background subtraction procedure: black squares correspond to the subtraction of the measurement on the Lo-Prec/Lo-dPMMA solution sample I_{exp} (inset, black circles) minus the solvent dDMF (inset, red circles). The green line is the performed RPA fit, and the red line is the constant background obtained from the high-Q fit, I_{inc}. The curves shown in this work are the blue squares, which correspond to the black squares minus the red line, and the black line is the background subtracted RPA fit.

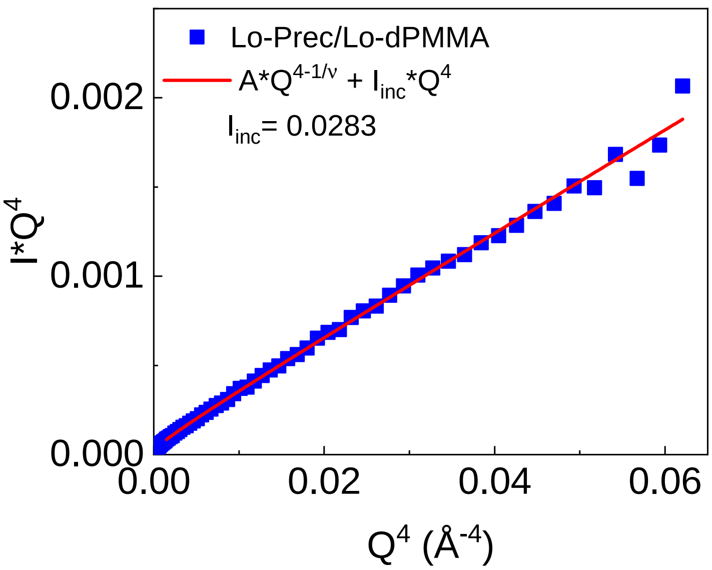

Figure S5: $I * Q^4$ vs Q^4 for Lo-Prec/Lo-dPMMA. This is an example of quantification of incoherent scattering, which is taken as the slope of the line. The fit is performed in the 0.2-0.5 Q-range.

samples, which would be indicative of compact objects, being more pronounced in the sample crowded with longer dPMMA chains (more compact objects). The data with no background subtraction is also shown for comparison. Even without background subtraction, it can be observed that the SCNPs crowded with Hi-dPMMA have a lower value of the scaling exponent, as can be deduced from the slope of the data around $Q \sim 0.1$ Å^{-1} (see Table 3).

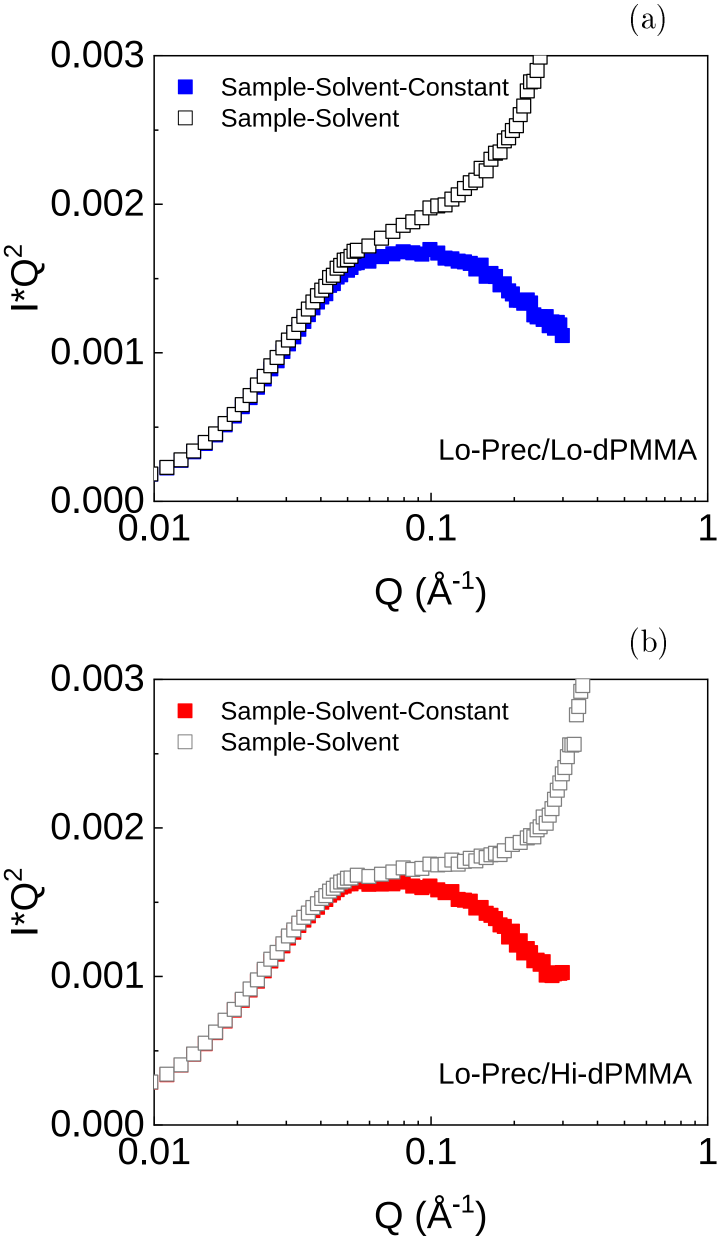

Figure S6: Kratky representation of the (a) Lo-Prec/Lo-dPMMA and (b) Lo-Prec/Hi-dPMMA SANS data with (solid symbols) and without solvent subtraction (empty symbols).

References

(S1) Moreno, A. J.; Verso, F. L.; Arbe, A.; Pomposo, J. A.; Colmenero, J. Concentrated Solutions of Single-Chain Nanoparticles: A Simple Model for Intrinsically Disordered Proteins under Crowding Conditions. *Journal of Physical Chemistry Letters* **2016**, *7*, 838–844.
